# Gene Expression Profile in Delay Graft Function: Inflammatory Markers Are Associated with Recipient and Donor Risk Factors

**DOI:** 10.1155/2014/167361

**Published:** 2014-05-19

**Authors:** Diego Guerrieri, Luis Re, Jorgelina Petroni, Nella Ambrosi, Roxana E. Pilotti, H. Eduardo Chuluyan, Domingo Casadei, Claudio Incardona

**Affiliations:** ^1^CEFYBO-School of Medicine, University of Buenos Aires, Paraguay 2155, 16th Floor, C1121ABG Buenos Aires, Argentina; ^2^Instituto de Nefrología de Buenos Aires, Cabello 3889, C1425APQ Buenos Aires, Argentina; ^3^GADOR S.A., Darwin 429, C1414CUH Buenos Aires, Argentina

## Abstract

*Background.* Delayed graft function (DGF) remains an important problem after kidney transplantation and reduced long-term graft survival of the transplanted organ. The aim of the present study was to determine if the development of DGF was associated with a specific pattern of inflammatory gene expression in expanded criteria of deceased donor kidney transplantation. Also, we explored the presence of correlations between DGF risk factors and the profile that was found. *Methods.* Seven days after kidney transplant, a cDNA microarray was performed on biopsies of graft from patients with and without DGF. Data was confirmed by real-time PCR. Correlations were performed between inflammatory gene expression and clinical risk factors. *Results.* From a total of 84 genes analyzed, 58 genes were upregulated while only 1 gene was downregulated in patients with DGF compared with no DGF (*P* = 0.01). The most relevant genes fold changes observed was IFNA1, IL-10, IL-1F7, IL-1R1, HMOX-1, and TGF-*β*. The results were confirmed for IFNA1, IL-1R1, HMOX-1 and TGF-*β*. A correlation was observed between TGF-*β*, donor age, and preablation creatinine, but not body mass index (BMI). Also, TGF-*β* showed an association with recipient age, while IFNA1 correlated with recipient BMI. Furthermore, TGF-*β*, IFNA1 and HMOX-1 correlated with several posttransplant kidney function markers, such as diuresis, ultrasound Doppler, and glycemia. *Conclusions.* Overall, the present study shows that DGF is associated with inflammatory markers, which are correlated with donor and recipient DGF risk factors.

## 1. Introduction

Delayed graft function (DGF) is a frequent event after kidney transplantation that strongly correlates with a lower graft survival rate [1]. Although there are several different definitions among transplant centers and in the literature [2], the most accepted definition of DGF is the need for dialysis within one week of transplantation. The reported incidence of DGF varies from 3.4% in living donor transplants to 31.2% in expanded criteria or 37.1% in donation after cardiac death donors [3]. However, the incidence is much higher in our Center (unpublished data) and in Latin-American Centers [4].

DGF is an independent risk factor for decreased graft survival. In the long-term, patients with DGF had a 49% pooled incidence of acute rejection compared to 35% incidence in non-DGF patients [1]. Several factors have been ascribed for the DGF occurrence, such as donor, recipient, and transplant procedural factors [5]. Among the first factors, increased age, hypertension, creatinine clearance, vascular sclerosis, weight, female gender, and nontraumatic death have been described. The recipient related factors are the presence of a sensitization state, the ethnicity, proinflammatory cytokines, and the mean arterial pressure.

Based on the strong association between the occurrence of DGF and the risk of acute rejection, great effort has been done to understand the pathogenesis, to identify the risk factors, and to find therapies that tend to diminish the incidence of DGF. Thus, several immunologic factors and coagulant mechanisms have been described that influence the development of DGF [6–8]. However, the cold ischemia time (CIT) seems to be one of the most important factors that influence the appearance of DGF [9, 10]. Unfortunately, in our country, the CIT is very high, that is, more than 24 hours. This is in agreement with the 75% incidence of DGF in our center. Therefore, the correct identification of the factors that influence DGF, it would benefit understanding the mechanisms responsible for the phenomenon.

In this study, we used a strategy to identify the influence that donor and recipient factors have on the inflammatory mechanisms of the DGF. We performed a microarray-based gene expression analysis and we examined the inflammatory markers on kidney biopsies of patients with and without DGF. Once the inflammatory markers were identified, correlations were performed with different donor and recipient DGF risk factors. We found that up- and downmodulated inflammatory markers were differentially correlated with singular donor and recipient risk factors.

## 2. Materials and Methods

### 2.1. Patients and Biopsies

Thirty four kidney transplanted patients were enrolled for these studies after giving written informed consent according to the Declarations of Helsinki. The clinical and research activities being reported are consistent with the Principles of the Declaration of Istanbul as outlined in the “Declaration of Istanbul on Organ Trafficking and Transplant Tourism”. Biopsies were obtained 7 days after transplant between December 2008 and June 2010. This study was approved by an Institutional Review Board.

Biopsies were obtained under ultrasound guidance by spring-loaded needles (ASAP Automatic Biopsy, Microvasive, Watertown, MA). Patients were grouped according to the presence of DGF. Posttransplant hemodialysis requirement was used to define DGF.  Table 1 shows the inclusion and exclusion criteria for patients included in this study and Table 2 shows the clinical characteristics of the patients enrolled for this study. All patients were treated with (i) induction therapy of thymoglobulin (7–14 days) and metilprednisolone (500 mg i.v.); (ii) maintenance immunosuppression with sirolimus (8–12 ng/mL), mycophenolate sodium (1440 mg), and prednisone (4 mg/day); (iii) prophylactic treatment of* Ganciclovir* IV (GCV-iv) 5 mg/kg/day or* Valganciclovir* (*VGCV*) 900 mg/day and trimethoprim-sulphamethoxazole (TMP-SMX).

### 2.2. Real-Time PCR Microarray Analysis

RNA was isolated by a phenol-based method from kidney biopsies by homogenization in 5 mL of TRIzol (Invitrogen, Carlsbad, CA). RNA was cleaned up with SABiosciences RT^2^-qPCR-Grade RNA isolation kit. The concentration and purity of RNA were determined by measuring the absorbance in a spectrophotometer. Sample dilutions were measured in 10 mM Tris at pH 8. Absorbance A260/A230 ratio was greater than 1.7 and A260/A280 was greater than 2.0 in all samples analyzed. Also an aliquot of each RNA sample was run on a denaturing agarose gel and sharp bands were present for both the 18S and 28S ribosomal RNA. Samples were discarded if signals of RNA degradation were observed in the agarose gel such as smearing or shoulders on the RNA peaks. RNA samples (1 *μ*g) were reverse-transcribed into cDNAs using a first-strand cDNA RT kit (SABioscience, CA). Then, samples were analyzed according to the manufacturer's recommendations using the “Innate & Adaptive Immune Responses” array in conjunction with the RT^2^ Profiler PCR Array System from SuperArray Bioscience (catalog number: PAHS-052Z, Frederick, MD). A total of 84 inflammatory related genes were examined (see Table 1 in Supplementary Material available online at http://dx.doi.org/10.1155/2014/167361). The array was initially performed with 16 RNA from kidney biopsies samples. For this, real-time PCR was performed using a 96-well format PCR array and an Applied Biosystems 7500 real-time PCR unit. Primers for all genes for real-time PCR of the microarray analysis had been pretested and confirmed by the manufacturer. Assay includes positive and negative controls as well as three sets of housekeeping genes for normalization purposes. Analysis of real-time PCR results is based on the ΔΔCt method with normalization of the raw data to housekeeping genes. Data were analyzed using the web-based PCR array data analysis software (SABiosciences). A 2-fold cut off threshold was used to define up or downmodulation of the genes analyzed.

### 2.3. Real-Time PCR

The result of the microarray was analyzed for confirmation by using a SYBR Green-based real-time PCR. Briefly, RNA samples from 11 no DGF and 23 DGF patients were tested for IL-1R1, IL-10, IL-1F7, IFNA1, HMOX-1, and TGF-*β* gene expression using the qPCR SuperMix Universal (Invitrogen, CA). Reaction solutions were prepared using reagents from the one-step SYBR Green Quantitative RT-PCR kit (Invitrogen, CA) combined with 0.25 *μ*M of each primer and 1 *μ*g of total RNA. The settings for the PCR instrument were as follows: 42°C for 30 min, 94°C for 2 min, and 40 cycles of 95°C for 15 s followed by 60°C for 1 min. Fluorescent signals were monitored sequentially for each sample tube once per cycle at the end of the elongation step. The specificity of the RT-PCR products was confirmed by analysis of melting curves and by omission of the reverse transcriptase. Human *β*-actin gene expression from the same RNA sample was also tested for normalization and quantification. The result was expressed as the fold expression normalized to *β*-actin. The gene expression was considered not detectable if the ratio of the gene against *β*-actin was smaller than 0.001. The data of the undetectable gene, was not plotted in Figures 3 and 4.

### 2.4. Statistical Analyses

For PCR array data analysis we used the SABiosciences RT^2^ Profiler Data Analysis Software to determine gene expression profiles (http://pcrdataanalysis.sabiosciences.com/pcr/arrayanalysis.php), which determined fold regulation values for each gene using the relative quantification 2-ΔΔCt method. ΔCt values were normalized using the mean values of three housekeeping genes: *β*-Actin, *β*-2-microglobulin, and GAPDH. All wells with a Ct value above 35 cycles were excluded from the analysis. This left 84 transcripts for analysis. Mann Whitney tests were used to compare means of continuous variables. Nonparametric test with Spearman's rank correlation coefficient was used for analyzing correlation. A *P* value <0.05 was considered significant. Graphs were generated by GraphPad Prism (GraphPad, Inc., La Jolla, CA).

## 3. Results

### 3.1. Microarray

**Figure 1:**
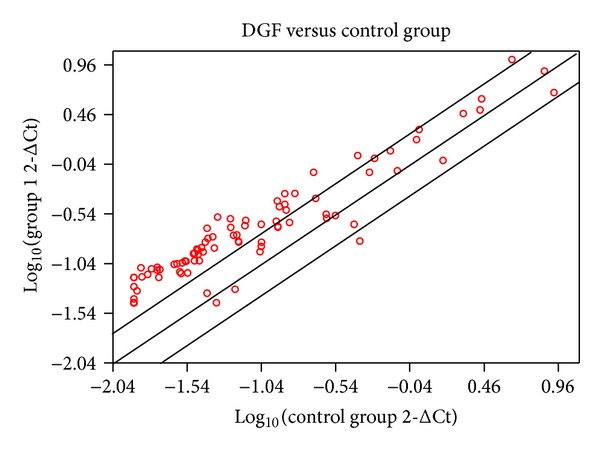
Scatter plot analysis of gene expression profiling on expanded criteria of kidney transplant patients. cDNA microarray analysis on 16 RNA samples from kidney transplant patients. After normalization on housekeeping genes, the final scores of each of the genes of all arrays were compared with those from the control array. The scatter plot was acquired as described in Materials and Methods. The *y*-axis represents log scores from DGF patients (group 1) and the *x*-axis represents log scores from no DGF patients (group 2). Each symbol represents one gene. Those gene outside the boundaries represent 2-fold higher or lower expressed in DGF patients.

**Figure 2:**
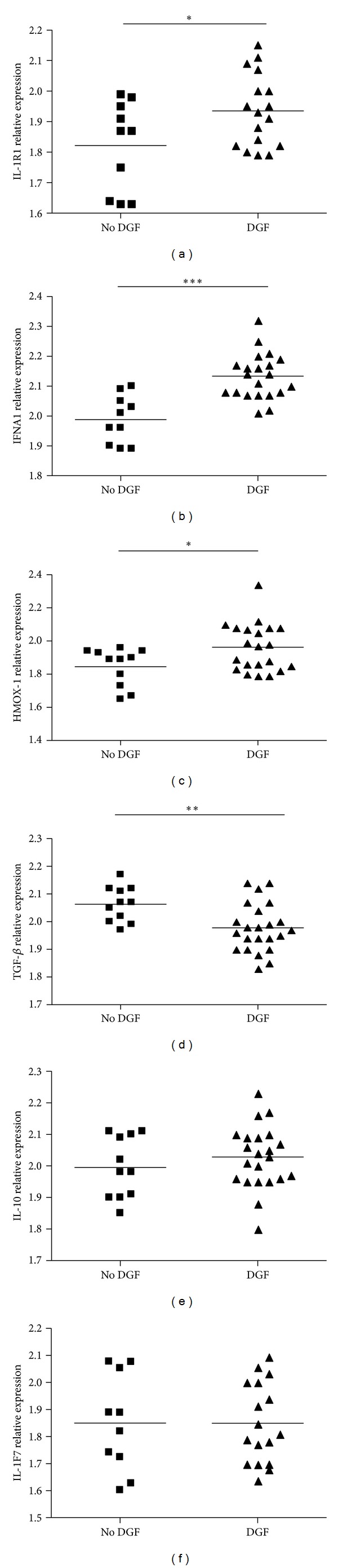
Real-time PCR results evaluating mRNA levels of inflammatory pathway related genes in kidney transplant patients. IL-1R1, IFNA1, HMOX-1, TGF-*β*, IL-10, and IL-1F7 were assayed by real-time PCR in whole kidney RNA samples from no DGF patients and DGF patients. Values were normalized to the level of *β*-actin RNA. Relative expression levels of all genes were calculated as 2^^^[(Ct reference gene) − (Ct target gene)]. *P < 0.05, **P < 0.01, and ***P < 0.001 by Mann Whitney test.

Quantitative real-time PCR microarray was used to analyze the gene expression profile in biopsy samples of 16 expanded criteria kidney transplant patients. We analyzed and compared the inflammatory genes profile between DGF (*n* = 8) and no DGF (*n* = 8) patients. Supplementary Table  2 shows a summary of the clinical characteristics of the patients enrolled for the microarray study. Seven day posttransplant kidney biopsies samples were used to perform the assay. From a total of 84 genes analyzed, 58 genes were upregulated while only 1 gene was downregulated in kidney biopsies from patients with DGF compared with no DGF (Figure 1). The most relevant genes upregulated, at least by two- or more-fold were IL-1R1, IL-10, IFNA1, IL-1F7, and HMOX-1 (Table 3). On the contrary, only TGF-*β* was downmodulated in DGF patients (Table 3). In order to confirm the results obtained with the microarray, we then performed real-time PCR for these genes. For this confirmation assay, we used a total of 34 biopsies samples (11 no DGF and 23 DGF patients). Although we were not able to detect some of the genes in all biopsies samples analyzed, the results obtained with the real-time PCR assay confirmed that IL-1R1 (Figure 2(a)), IFNA1 (Figure 2(b)) and HMOX-1 (Figure 2(c)) genes were upregulated and TGF-*β* (Figure 2(d)) gene was downregulated in DGF patients (Figure 1, *P* < 0.01). However, we were unable to show statistical differences in IL-10 (Figure 2(e)) and IL-1F7 (Figure 2(f)) genes between groups of patients analyzed.

### 3.2. Correlations between Gene Expression and Clinical Features

To further determine if the changes in gene expression observed in the biopsies could be related to donor specific characteristics, CIT, or recipients features we analyzed correlations between genes expression and clinical parameters.

**Figure 3:**
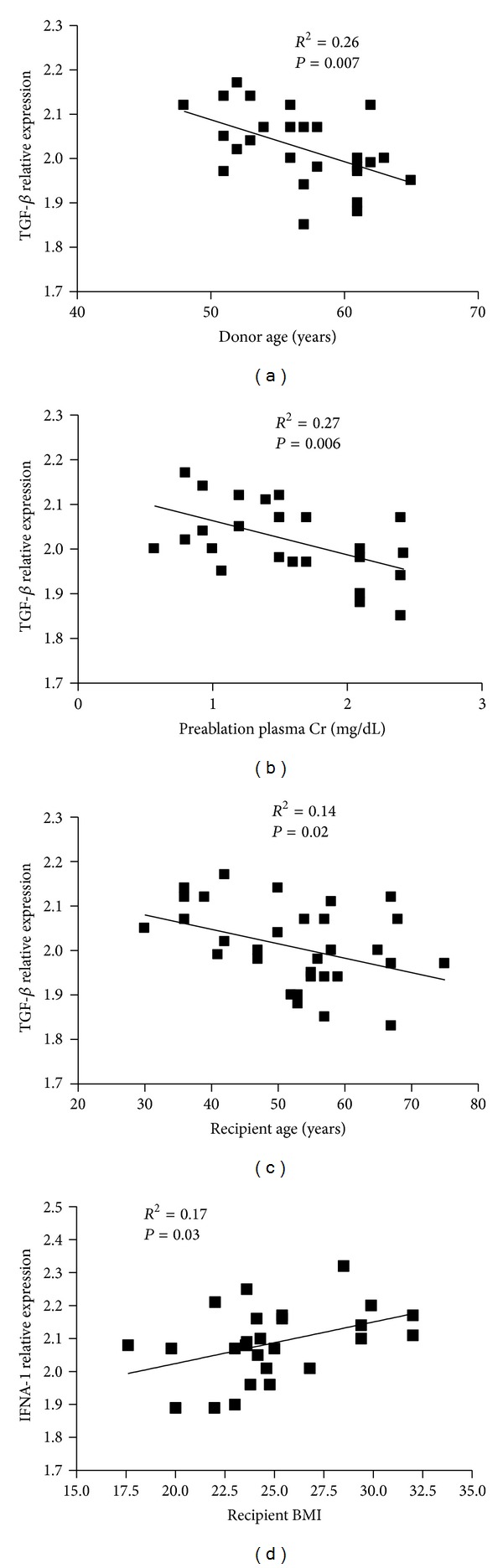
Correlation between the gene expression levels and recipient and donor risk factors. Correlation between donor age and TGF-*β* (a), preablation creatinine and TGF-*β* (b), recipient age and TGF-*β* (c), and recipient BMI and IFNA-1 (d). Regression lines are shown in each figure with correlation coefficients (*R* ^2^) and *P* values.

**Figure 4:**
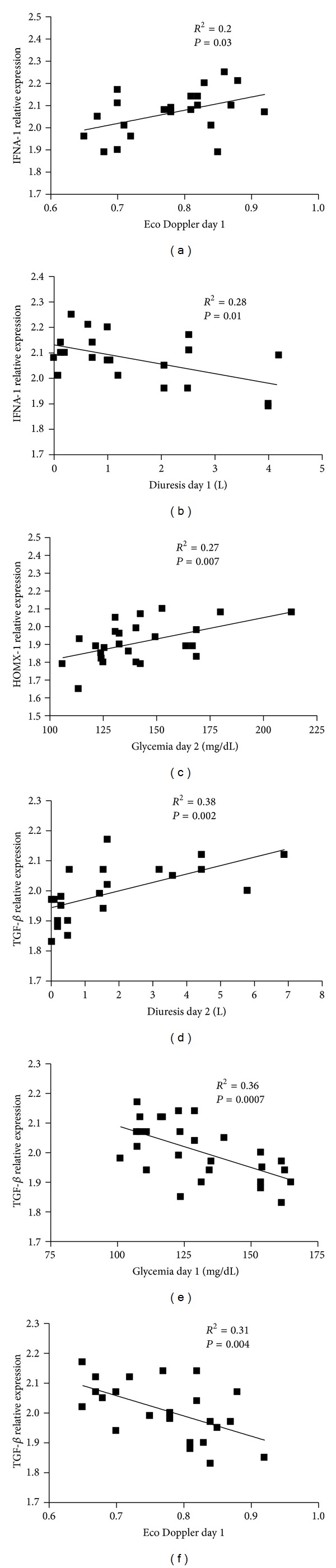
Correlation between the gene expression levels and kidney functional parameters. Correlation between Eco Doppler index of day 1 and IFNA1 (a), diuresis of day 1 and IFNA1 (b), glycemia of day 2 and HMOX-1 (c), diuresis of day 2 and TGF-*β* (d), glycemia of day 1 and TGF-*β* (e), and Eco Doppler index of day 1 and TGF-*β* (f). Regression lines are shown in each figure with correlation coefficients (*R* ^2^) and *P* values.

The donor specific characteristics analyzed were the age, preablation creatinine and the body mass index (BMI). Correlations were performed with the genes that were up- and downmodulated. We did not find any correlation with donor BMI. However, we found a correlation between TGF-*β* gene expression with donor age (Figure 3(a)) and preablation creatinine (Figure 3(b)). Then, the correlations were analyzed between CIT and genes expression. Although, we did not find statistical significant correlations between this risk factor and inflammatory markers, we found a trend between IFNA1 and CTI (*P* = 0.057; data not shown). Following this, the analysis was performed with recipient characteristics, such as age, BMI, and time on dialysis. We did not find correlations with time on dialysis. However, we found that TGF-*β* expression inversely correlated with recipient age (Figure 3(c)). Also, there was a positive correlation between IFNA1 and recipient BMI (Figure 3(d)). Finally, the correlations were examined with markers of kidney function during the first two days after the transplant. When IFNA1 was analyzed, a positive and negative correlation was observed with the Doppler ultrasound resistive index and diuresis, respectively (Figures 4(a) and 4(b)). Furthermore, for HMOX-1 a positive correlation was observed with glycemia (Figure 4(c)). Finally, for TGF-*β* we found a positive correlation with diuresis (Figure 4(d)) and the amount of units of insulin administered (*P* = 0,001; data not shown) to the patients, while negative correlation was observed with glycemia (Figure 4(e)) and Doppler ultrasound resistive index (Figure 4(f)).

## 4. Discussion

Sometimes a biopsy is performed at the time of transplantation in order to check for preexisting lesions in the donor kidney. However, it has not been helpful as an indicator for therapeutic intervention. Recent findings suggest the importance of recipient factors in addition to the donor factors [5]. In this study we performed a 7 day posttransplant biopsy that should reflect both donor and recipient factors, although the interpretation of the data requires a comprehensive analysis because multiple factors are involved.

A systematic biological assessment of these molecular changes suggests that the inflammatory process driven by ischemia reperfusion injury (IRI) is largely responsible for DGF occurrence. The analysis of the microarray shows that several inflammatory mediators are upregulated in DGF patients. The most significant mediators but not the only ones were IL-R1, IL-10, IFNA1, IL-1F7, and HMOX-1. However, only IFNA1, IL-1R1, and HMOX-1 were confirmed to differ between DGF and no DGF group by real-time PCR. It is important to mention that the partial selection of the mediators analyzed for confirmation with the real-time PCR was chosen based on the fold changes that we found in the microarray.

It is known that drugs used for immunosuppression may affect DGF and gene expression profile [11]. For example, it has been described that sirolimus prolongs recovery from delayed graft function after cadaveric renal transplantation [12]. In our study, biopsies were obtained before the introduction of sirolimus in the immunosuppressive regime. Therefore, gene expression data was not influenced by sirolimus. It is important to mention that in this study, the differential gene expression profile between DGF and no DGF patients was not influenced by immunosuppression drugs, since all patients received the same immunosuppressive regime.

Based on the function of each IFNA1 and IL-1R1, we can speculate that the pattern of cytokines upregulated was compatible with a proinflammatory state. For example, IFNA1 has a well-known antiviral action and also plays a major role in the adaptive immune response acting on dendritic cells, NK cells, and lymphocytes [13, 14]. Furthermore, IL-1R1 is a signaling receptor for IL-1. This cytokine is a potent factor with pleiotropic functions such as stimulating angiogenesis at inflamed tissue sites, triggering proinflammatory cytokine and contributing to the polarization of Th17 cells [15]. The upregulation of IL-1R1 observed in biopsies of DGF patients suggests that this kidney may be more suitable to respond under inflammatory microenvironments, where the IL-1 is present.

In agreement with this proinflammatory microenvironment, we found that TGF-*β* is downmodulated in DGF patients. TGF-*β* is a 25-kDa homodimeric peptide with pleiotropic activity on different cell types [16]. Their immunosuppressive effects are known by inhibiting lymphocyte activation, but also proinflammatory activity has been described [16, 17]. Moreover, recent studies suggest that high levels of TGF-*β* activated T cells that cause cytotoxic damage and acute rejection [18]. However, also a low TGF-*β* production in both the donor and recipient was associated with risk for early rejection and worse graft function at 4 years [18].

HMOX-1 is another mediator that is upregulated in DGF patients. HMOX-1 is a very important enzyme which degrades heme into carbon monoxide, biliverdin, and free iron [19, 20]. The expression of HMOX-1 is inducible in response to pathophysiological stresses and it has antioxidant, anti-inflammatory, and antiapoptotic activity [19, 20]. In fact, the expression of HMOX-1 protects from the induction of chronic allograft rejection [21, 22]. Also, it has been described that TGF-*β* induces HMOX-1 [23]. However, since TGF-*β* is downmodulated in our study, we believe that HMOX-1 upregulation is due to the proinflammatory microenvironment. Perhaps, the high levels of HMOX-1 may act as feedback mechanisms that tend to control the proinflammatory state.

Differences between no DGF and DGF groups were also seen in long-term outcome such as patients' survival and graft function (data not shown), as was expected. At three years after kidney transplant, 91% of the patients of the no DGF group were alive and with a good kidney function; however with the no DGF group 74% were alive with normal kidney function.

The findings in biopsies taken at seven days are the results of factors contributed by the donor (such as age, BMI, and preablation creatinine), the procurement period (CIT, preservation liquid), the receptor (age, BMI), and early posttransplant period (ischemia reperfusion injury). In fact, there are much more factors that influence the DGF, such as immunosuppression therapy [11]. The relative impact of each of the markers on DGF is difficult to assess. However, by using the inflammatory markers as the endpoint and performing the correlations with each of the factors that contributed to the DGF, we determined the relative strength of each factor on the inflammatory process. Of the donor derived factors analyzed, donor age and preablation creatinine were associated with TGF-*β*. Since preablation, creatinine reflects the state of the kidney to be engrafted; this association with TGF-*β* could be expected. Surprisingly, we did not find a significant correlation with CIT as was described in [24], but we did find a trend with IFNA1 (*P* = 0,057; data not shown). Perhaps, the fact that there was not difference in CIT between groups (Table 2) underscores this result. Of the recipient derived factors, the age was associated with TGF-*β*, while the BMI with IFNA-1. Thus, recipient factors act both on pro- and anti-inflammatory mediators. Based on the link between adipose tissue and inflammation [25] we can speculate that obesity influences more over the proinflammatory markers, while the recipient age decreases the anti-inflammatory marker. The result of the recipient factors is to shift the balance to a proinflammatory state. However, it seems that the donor factors influence the inflammation by decreasing the immunosuppressive cytokine, such as TGF-*β*.

Finally, we found more correlations at the posttransplant period, more precisely with kidney function markers. Some of them may be ascribed to the IRI. For example, the association between IFNA-1 with diuresis and ultrasound Doppler, HMOX-1 with and glycemia, and TGF-*β* with diuresis, ultrasound Doppler, glycemia, and units of insulin administration might represent the sum of the factors derived from the donor and the recipient plus the reperfusion injury.

Gene array has been used before in kidney transplantation [26, 27]. Most of them were used to monitor the graft status, infection, or graft rejections. Despite the high cost of the technique, by performing the gene array we may detect biomarkers that allow us to predict the outcome of the graft.

## 5. Conclusion

Our results identify inflammatory molecular changes in kidney transplant of DGF patients that associates with clinical risk factors. The strength of these factors on the inflammatory process is uneven. Overall, these results suggest for the first time that changes in some inflammatory mediators in kidney transplantation recipients may be ascribed to donors while others to the recipients' characteristics.

## Supplementary Material

Supplementary Table 1: The RT^2^ Profiler^TM^ PCR Array profiles the expression of 84 genes involved in the host Innate & Adaptive Immune responses. This array includes genes related to the IL-1R, Toll-like Receptor (TLR) Signaling Pathways, acute-phase response, complement activation, and the inflammatory response. Genes involved in the innate immune response and septic shock are also included on this array.
Supplementary Table 2: A summary of the clinical characteristics of the patients enrolled only for the microarray study is shown. The “Innate & Adaptive Immune Responses” array was performed with 8 DGF and 8 no DGF patients.

## Figures and Tables

**Table 1:** Inclusion and exclusion criteria.

Inclusion criteria	Exclusion criteria
*Donors *	*Donors *
>60 years old or between 50 and 59 years who fulfilled at least 2 of the following criteria. (i) History of hypertension (ii) Stroke as cause of death (iii) Preablation sCr >1.5 mg/dL	(i) IV drugs abuse (ii) HIV positive (iii) Kidneys from standard donors
*Recipients *	*Recipients *
(i) First disease donor kidney transplant (ii) >18 years (iii) Signed informed consent (iv) Panel reactive antibody < 20%	(i) Diabetes mellitus (ii) Chronic use of steroids (iii) Pregnant women/lactancy period (iv) History of cancer or linfoproliferative disorder

**Table 2:** Characteristics of renal transplant patients.

	Group 1 (DGF, *n* = 23)	Group 2 (No DGF, *n* = 11)	*P* values
Recipient age (years)	54.7 ± 8.2	50.1 ± 16.1	0.27
Recipient BMI	25.7 ± 4.3	23.5 ± 1.3	0.10
Time of dialysis (years)	4.8 ± 3.9	5.67 ± 2.7	0.51
HLA MM (A, B, DR)	3 ± 1.7	3.4 ± 1.1	0.48
CIT (hours)	25.2 ± 3	23.9 ± 5.5	0.37
Donor age (years)	56.4 ± 4.8	56.8 ± 4.6	0.81
Donor BMI	26.9 ± 4.2	27.9 ± 5.3	0.55
Preablation creatinine (mg/dL)	1.8 ± 0.7	1.3 ± 0.3	0.03
Day 1 diuresis (L)	0.37 ± 0.5	2.7 ± 2.5	0.0001
Day 1 Eco Doppler	0.81 ± 0.6	0.73 ± 0.09	0.66
Day 1 uremia (mg/dL)	126.5 ± 27.4	119.1 ± 26.9	0.46
Day 1 creatinine(mg/dL)	7.62 ± 2	9.08 ± 1.5	0.039
Day 1 serum Na^+^ (mEq/L)	136.4 ± 3	135.2 ± 2.8	0.27
Day 2 diuresis (L)	0.45 ± 0.56	3.2 ± 2.3	0.0001
Day 2 uremia (mg/dL)	142.9 ± 28	137.9 ± 31.2	0.64
Day 2 creatinine (mg/dL)	8.05 ± 2.2	8.52 ± 1.1	0.52
Day 2 serum Na^+^ (mEq/L)	135.7 ± 2.7	136.2 ± 2.4	0.60

BMI: body mass index; HLA MM: human leukocyte antigen mismatch.

**Table 3:** Real-time PCR microarray: genes up- and downmodulated in kidney biopsies from DGF versus no DGF patients.

	Mediators	Fold change
Upregulation	IL-1R1	4.9
	IL-10	4.8
	IFNA1	4.4
	IL-1F7	4.3
	HMOX-1	2.9

Downregulation	TGF-β	3.3

## Abbreviations

BMI: Body mass index
CIT: Cold ischemia time
DGF: Delayed graft function
HMOX-1: Heme oxygenase 1
IFNA1: Interferon alpha 1
IL-1F7: Interleukin1 family member 7
IL-1R1: Interleukin 1 receptor, type I
IL-10: Interleukin 10
IRI: Ischemia reperfusion injury
TGF-*β*: Transforming growth factor Beta.
