# Supplementary material for: Gene Expression Profile in Delay Graft Function: Inflammatory Markers Are Associated with Recipient and Donor Risk Factors
Source: Mediators Inflamm. 2014 May 19;2014:167361. doi: 10.1155/2014/167361 (PMC4052172; doi:10.1155/2014/167361)
Supplement: Supplementary file 1 — Supplementary Table 1: The RT2 ProfilerTM PCR Array profiles the expression of 84 genes involved in the host Innate & Adaptive Immune responses. This array includes genes related to the IL-1R, Toll-like Receptor (TLR) Signaling Pathways, acute-phase response, complement activation, and the inflammatory response. Genes involved in the innate immune response and septic shock are also included on this array. Supplementary Table 2: A summary of the clinical characteristics of the patients enrolled only for the microarray study is shown. The “Innate & Adaptive Immune Responses” array was performed with 8 DGF and 8 no DGF patients. [file 167361.f1.doc]

Supplementary data. Table 1.

| **Position** | **Symbol** | **Description** |
| --- | --- | --- |
| A01 | APCS | Amyloid P component, serum |
| A02 | C3 | Complement component 3 |
| A03 | CASP1 | Caspase 1, apoptosis-related cysteine peptidase (interleukin 1, beta, convertase) |
| A04 | CCL2 | Chemokine (C-C motif) ligand 2 |
| A05 | CCL5 | Chemokine (C-C motif) ligand 5 |
| A06 | CCR4 | Chemokine (C-C motif) receptor 4 |
| A07 | CCR5 | Chemokine (C-C motif) receptor 5 |
| A08 | CCR6 | Chemokine (C-C motif) receptor 6 |
| A09 | CCR8 | Chemokine (C-C motif) receptor 8 |
| A10 | CD14 | CD14 molecule |
| A11 | CD4 | CD4 molecule |
| A12 | CD40 | CD40 molecule, TNF receptor superfamily member 5 |
| B01 | CD40LG | CD40 ligand |
| B02 | CD80 | CD80 molecule |
| B03 | CD86 | CD86 molecule |
| B04 | CD8A | CD8a molecule |
| B05 | CRP | C-reactive protein, pentraxin-related |
| B06 | CSF2 | Colony stimulating factor 2 (granulocyte-macrophage) |
| B07 | CXCL10 | Chemokine (C-X-C motif) ligand 10 |
| B08 | CXCR3 | Chemokine (C-X-C motif) receptor 3 |
| B09 | DDX58 | DEAD (Asp-Glu-Ala-Asp) box polypeptide 58 |
| B10 | FASLG | Fas ligand (TNF superfamily, member 6) |
| B11 | FOXP3 | Forkhead box P3 |
| B12 | GATA3 | GATA binding protein 3 |
| C01 | HLA-A | Major histocompatibility complex, class I, A |
| C02 | HLA-E | Major histocompatibility complex, class I, E |
| C03 | ICAM1 | Intercellular adhesion molecule 1 |
| C04 | IFNA1 | Interferon, alpha 1 |
| C05 | IFNAR1 | Interferon (alpha, beta and omega) receptor 1 |
| C06 | IFNB1 | Interferon, beta 1, fibroblast |
| C07 | IFNG | Interferon, gamma |
| C08 | IFNGR1 | Interferon gamma receptor 1 |
| C09 | IL10 | Interleukin 10 |
| C10 | IL13 | Interleukin 13 |
| C11 | IL17A | Interleukin 17A |
| C12 | IL18 | Interleukin 18 (interferon-gamma-inducing factor) |
| D01 | IL1A | Interleukin 1, alpha |
| D02 | IL1B | Interleukin 1, beta |
| D03 | IL1R1 | Interleukin 1 receptor, type I |
| D04 | IL2 | Interleukin 2 |
| D05 | IL23A | Interleukin 23, alpha subunit p19 |
| D06 | IL4 | Interleukin 4 |
| D07 | IL5 | Interleukin 5 (colony-stimulating factor, eosinophil) |
| D08 | IL6 | Interleukin 6 (interferon, beta 2) |
| D09 | IL8 | Interleukin 8 |
| D10 | IRAK1 | Interleukin-1 receptor-associated kinase 1 |
| D11 | IRF3 | Interferon regulatory factor 3 |
| D12 | IRF7 | Interferon regulatory factor 7 |
| E01 | ITGAM | Integrin, alpha M (complement component 3 receptor 3 subunit) |
| E02 | JAK2 | Janus kinase 2 |
| E03 | LY96 | Lymphocyte antigen 96 |
| E04 | LYZ | Lysozyme |
| E05 | MAPK1 | Mitogen-activated protein kinase 1 |
| E06 | MAPK8 | Mitogen-activated protein kinase 8 |
| E07 | MBL2 | Mannose-binding lectin (protein C) 2, soluble |
| E08 | MPO | Myeloperoxidase |
| E09 | MX1 | Myxovirus (influenza virus) resistance 1, interferon-inducible protein p78 (mouse) |
| E10 | MYD88 | Myeloid differentiation primary response gene (88) |
| E11 | NFKB1 | Nuclear factor of kappa light polypeptide gene enhancer in B-cells 1 |
| E12 | NFKBIA | Nuclear factor of kappa light polypeptide gene enhancer in B-cells inhibitor, alpha |
| F01 | NLRP3 | NLR family, pyrin domain containing 3 |
| F02 | NOD1 | Nucleotide-binding oligomerization domain containing 1 |
| F03 | NOD2 | Nucleotide-binding oligomerization domain containing 2 |
| F04 | RAG1 | Recombination activating gene 1 |
| F05 | RORC | RAR-related orphan receptor C |
| F06 | SLC11A1 | Solute carrier family 11 (proton-coupled divalent metal ion transporters), member 1 |
| F07 | STAT1 | Signal transducer and activator of transcription 1, 91kDa |
| F08 | STAT3 | Signal transducer and activator of transcription 3 (acute-phase response factor) |
| F09 | STAT4 | Signal transducer and activator of transcription 4 |
| F10 | STAT6 | Signal transducer and activator of transcription 6, interleukin-4 induced |
| F11 | TBX21 | T-box 21 |
| F12 | TICAM1 | Toll-like receptor adaptor molecule 1 |
| G01 | TLR1 | Toll-like receptor 1 |
| G02 | TLR2 | Toll-like receptor 2 |
| G03 | TLR3 | Toll-like receptor 3 |
| G04 | TLR4 | Toll-like receptor 4 |
| G05 | TLR5 | Toll-like receptor 5 |
| G06 | TLR6 | Toll-like receptor 6 |
| G07 | TLR7 | Toll-like receptor 7 |
| G08 | TLR8 | Toll-like receptor 8 |
| G09 | TLR9 | Toll-like receptor 9 |
| G10 | TNF | Tumor necrosis factor |
| G11 | TRAF6 | TNF receptor-associated factor 6 |
| G12 | TYK2 | Tyrosine kinase 2 |

**Supplementary data. Table 2.**

|  | ***Group 1***  ***(DGF, n=8)*** | ***Group 2***  ***(No DGF, n=8)*** | ***P values*** |
| --- | --- | --- | --- |
| **Recipient Age (years)** | 51.5 ± 8.4 | 47.4 ± 17.8 | 0.53 |
| **Recipient BMI** | 25.5 ± 4 | 23.1 ± 1.8 | 0.14 |
| **Time of dialysis (years)** | 5.2 ± 3.9 | 4.6 ± 1.9 | 0.7 |
| **HLA MM (A, B, DR)** | 3.4 ± 1.4 | 3.2 ± 1.3 | 0.17 |
| **CIT (Hours)** | 24.5 ± 5.7 | 21.3 ± 3.2 | 0.18 |
| **Donor Age (years)** | 56.1 ± 4.1 | 54.8 ± 4 | 0.53 |
| **Donor BMI** | 29.2 ± 5.5 | 28 ± 5.6 | 0.67 |
| **Preablation Creatinine (mg/dl)** | 1.8 ± 0.9 | 1.1 ± 0.3 | 0.06 |
| **Day 1 Diuresis (L)** | 0.39 ± 0.5 | 3.5 ± 2.8 | 0.007 |
| **Day 1 Eco Doppler** | 0.8 ± 0.1 | 0.7 ± 0.1 | 0.06 |
| **Day 1 Ureamia (mg/dl)** | 135.3 ± 23.5 | 121.2 ± 35.4 | 0.36 |
| **Day 1 Creatinine(mg/dl)** | 8.4 ± 1.6 | 9 ± 1.1 | 0.39 |
| **Day 1 Serum Na^+^ (mEq/L)** | 136.3 ± 3.1 | 135.6 ± 2.4 | 0.62 |
| **Day 2 Diuresis (L)** | 0.44 ±0.65 | 2.6 ± 1.7 | 0.005 |
| **Day 2 Ureamia (mg/dl)** | 158.6 ± 49.3 | 139 ± 34 | 0.37 |
| **Day 2 Creatinine (mg/dl)** | 9± 2.3 | 8 ± 1 | 0.27 |
| **Day 2 Serum Na^+^ (mEq/L)** | 134.4 ± 2.3 | 136.2 ± 2.4 | 0.15 |

**BMI=Body Mass Index; HLA MM= Human Leukocyte Antigen Mismatch**
